# Effectiveness of a structured physiotherapy intervention on psychomotor and quality of life in children with autism spectrum disorder: A randomised controlled trial protocol^☆^

**DOI:** 10.1016/j.mex.2026.103873

**Published:** 2026-03-18

**Authors:** Nazurah Alwi, Asfarina Zanudin, Mahadir Ahmad, Nor Azizah Mohamad

**Affiliations:** a Physiotherapy Programme, Centre for Rehabilitation and Special Needs Studies, Faculty of Health Science, Universiti Kebangsaan Malaysia, Jalan Raja Muda Abdul Aziz, 50300, Kuala Lumpur, Wilayah Persekutuan Kuala Lumpur, Malaysia; b Clinical Psychology Programme, Centre for Community Health Studies, Faculty of Health Sciences, Universiti Kebangsaan Malaysia, Jalan Raja Muda Abdul Aziz, 50300, Kuala Lumpur, Wilayah Persekutuan Kuala Lumpur, Malaysia; c Physiotherapy Unit, Department of Rehabilitation, UKM Specialist Children’s Hospital, Jalan Yaacob Latif, Bandar Tun Razak, Cheras, 56000 Kuala Lumpur, Malaysia

**Keywords:** Autism spectrum disorder, Physiotherapy, Motor-based intervention, Psychomotor, Quality of life

## Abstract

Children with Autism Spectrum Disorder (ASD) frequently experience psychomotor impairments, limited participation in physical activities, and reduced quality of life (QoL). Exercise shows promising benefits, but physiotherapy approaches in ASD are underreported, with limited evidence supporting a structured framework that systematically targets motor and functional outcomes. This study protocol describes a single-blinded randomised controlled trial evaluating the effectiveness of a structured physiotherapy intervention on motor skills, physical activity levels, and behavioural and QoL among children with mild to moderate ASD. This trial will include 64 children aged 6–10 years with ASD. Participants will be randomly assigned to an intervention group or a control group. Both groups will receive usual care therapy, equated between groups. The intervention group will also receive structured physiotherapy twice weekly for 12 weeks, each session lasting 60 min. The intervention integrates coordination, balance, strength, and endurance training. Outcome assessments will be conducted at baseline and post-intervention using validated instruments: GARS-3, BOT-2, GLTEQ, CBCL and PedsQL. Data analysis will follow the intention-to-treat principle and will examine between-group and within-group changes across all outcome measures. Qualitative data will be analysed using thematic analysis to characterise themes reflecting participants' engagement and parents' and caregivers' perspectives on the intervention.

## Graphical abstract

**Figure:**
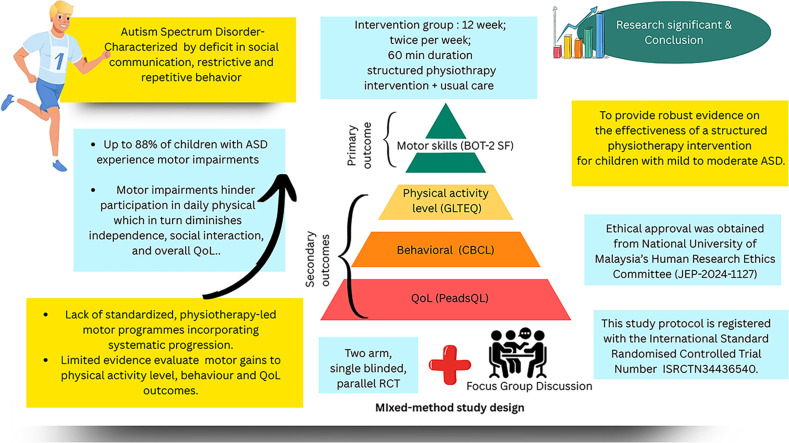

## Specifications table

**Table:** 

**Subject area**	Medicine and Dentistry
**More specific subject area**	Rehabilitation
**Name of your protocol**	Structured Physiotherapy Intervention
**Reagents/tools**	Gilliam Autism Rating Scale–3 (GARS-3), Bruininks-Oseretsky Test of Motor Proficiency–2 (BOT-2), Godin-Shephard Leisure-Time Exercise Questionnaire (GLTEQ), Child Behaviour Checklist (CBCL) and Pediatric Quality of Life Inventory (PedsQL).
**Experimental design**	This study employs a mixed-methods design integrating a randomised controlled trial (RCT) with semi-structured qualitative interviews involving children with ASD aged 6–10 years and their parents or caregivers.
**Trial registration**	This study protocol is registered with the International Standard Randomised Controlled Trial Number under the registration number ISRCTN34436540.
**Ethics**	Ethical approval was obtained from the National University of Malaysia’s Human Research Ethics Committee (JEP–2024–1127)
**Value of the Protocol**	Given the inconclusive results from the previous study, this protocol aims to provide essential evidence on the effectiveness of structured physiotherapy intervention by utilising robust outcome measures, including psychomotor and quality of life (QoL) among children with ASD. This study protocol will offer valuable guidance for clinicians on incorporating structured physiotherapy intervention into management for children with ASD, potentially enhancing therapeutic outcomes and improving patient care.

## Background

Autism Spectrum Disorder (ASD) affects approximately 1 in 36 children globally and represents a growing public health concern [1]. ASD is characterised by persistent difficulties in social communication and restricted, repetitive behaviours [2]. Beyond these core diagnostic characteristics, children with ASD commonly demonstrate motor impairments, including poor coordination, impaired balance, and delayed fine and gross motor skill development [3]. Evidence indicates that 80–88 % of children with ASD present motor impairments of clinical significance [4,5]. These motor impairments interfere with age-appropriate physical play, school-related movement tasks, and sport participation, which may reduce independence, limit peer interaction, and contribute to poorer overall quality of life (QoL) [6,7].

Neurobiological evidence supports the need for targeted motor rehabilitation [8]. Motor impairments in ASD are associated with altered cerebellar–cortical connectivity, contributing to deficits in balance control and motor planning [8]. Neuroimaging studies demonstrate reduced cerebellar volume and disrupted signalling between the cerebellum and the primary motor cortex, impairing implicit motor learning, a process typically acquired automatically through play in typically developing children [9]. Consequently, children with ASD more consistently benefit from structured programmes involving high repetition and explicit feedback, rather than unstructured motor practice alone [10].

A wide range of motor and exercise-based interventions has been evaluated in children with ASD, including fundamental motor skill programmes, yoga, rhythmic movement, aquatic therapy, exergaming, physical education, and general physical activity initiatives [[11], [12], [13]]. While these interventions show promising results, the evidence remains heterogeneous, with substantial variability in duration, frequency, supervision, instructor expertise, and progression criteria, limiting comparability and clarity about optimal treatment parameters [[11], [12], [13]]. Many programmes examine motor outcomes in isolation without evaluating broader functional pathways or QoL [14,15]. This leaves limited clarity about whether motor improvements translate into physical activity participation, behavioural gains, or QoL changes [[13], [14], [15]].

Despite its clinical relevance, physiotherapy remains underutilised in ASD management [7]. Physiotherapy provides an impairment-focused, clinically-led approach, applying motor learning strategies with task-oriented progression to improve movement quality and participation [16]. However, existing service utilisation statistics report high access to speech or occupational therapy, while fewer receive physiotherapy despite prevalent motor challenges [17]. Physiotherapy-led interventions for children with ASD remain poorly standardised and commonly consist of small or uncontrolled studies, limiting scalability or clinical translation [17,18]. Contributing factors include ASD being primarily perceived as a behavioural condition, limited specialist physiotherapy training, and low integration of physiotherapists within multidisciplinary care pathways [17,19].

This study protocol describes a single-blind RCT designed to evaluate a structured, progression-based physiotherapy-led motor intervention targeting motor impairments in children aged 6–10 years with mild to moderate ASD, assessed alongside physical activity, behaviour, and QoL. The trial includes caregiver-partnered interaction and a mixed-method evaluation to support implementation relevance. Evidence from this trial is expected to provide more precise clinical parameters and a replicable physiotherapy-led protocol. This may strengthen standardised motor-based intervention and support more consistent integration within existing ASD care pathways.

## Description of protocol

### Trial design

This study employs a mixed-methods research design, integrating a two-arm, parallel-group, single-blind randomised controlled trial (RCT) with semi-structured qualitative interviews involving children with ASD aged 6–10 years and their parents or caregivers. The primary aim is to evaluate the effectiveness of a structured physiotherapy intervention on psychomotor and QoL in children with mild to moderate ASD. The trial protocol was designed in accordance with the Consolidated Standards of Reporting Trials (CONSORT) 2010 and SPIRIT guidelines to ensure methodological rigour and transparency in reporting. This trial has been registered with the International Standard Randomised Controlled Trial Number under the registration number ISRCTN34436540.

### Study location

This multi-site trial will be conducted across two regions in Peninsular Malaysia, namely the East Coast Region in Terengganu and the Central Region in Kuala Lumpur. Participants will be recruited from community-based rehabilitation centres that provide services for children with ASD, operated in the two regions under the Ministry of Welfare, Malaysia. In Terengganu, recruitment will involve centres in three districts: Marang, Kuala Nerus, and Kuala Terengganu. In Kuala Lumpur, participants will be recruited from similar community-based centres and the Universiti Kebangsaan Malaysia (UKM) Specialist Children's Hospital. The intervention will be implemented at two primary sites: (1) the Physiotherapy Clinic, Faculty of Health Sciences, UKM, Kuala Lumpur, for participants from Kuala Lumpur, and (2) the Terengganu Autism Organisation Centre for participants from Terengganu.

### Sample size calculation

The sample size for this study was calculated using G*Power software version 3.1.9.4, employing an F-test for repeated-measures analysis of variance (ANOVA) with a within–between interaction [20]. The calculation was based on an alpha level of 0.05, a statistical power of 80 % (1–β = 0.80) and an effect size (f) of 0.12. This effect size was derived from the previous study [21] which investigated motor skill outcomes using the BOT-2 which is the primary outcome measure in the present study. The Cohen’s d reported in the previous study [21] was converted to Cohen’s f using the online tool provided by https://www.escal.site/. Based on these parameters, the minimum required sample size was calculated to be 58 participants. Additionally, accounting for an anticipated dropout rate of 10 % the required sample size is 64 participants, with 32 participants allocated to each group.

### Inclusion and exclusion criteria

Participants will be selected based on specific inclusion and exclusion criteria to ensure the sample is relevant. The inclusion criteria are as follows: (i) aged between 6 and 10 years old, (ii) have a confirmed diagnosis of ASD according to the DSM-5-TR, (iii) classified as having mild to moderate severity based on the GARS-3 autism index (55–100; level 1–2). (iv) demonstrate motor difficulties on the BOT-2 Short Form (iv) able to follow simple verbal instructions (v) have written informed consent provided by a parent or legal guardian. The exclusion criteria include (i) having sensory or physical impairments that would limit their ability to participate in the intervention, (ii) other neurological or developmental conditions such as epilepsy or cerebral palsy, or if they have physical disabilities arising from head injury or other acquired conditions, (iii) having co-occurring diagnosis such as Attention Deficit Hyperactivity Disorder and Down Syndrome, (iv) currently involved in another clinical trial or intensive motor programme, and (v) unstable medical conditions or recent changes in medical treatment.

### Participant screening, recruitment and randomisation

**Fig. 1:**
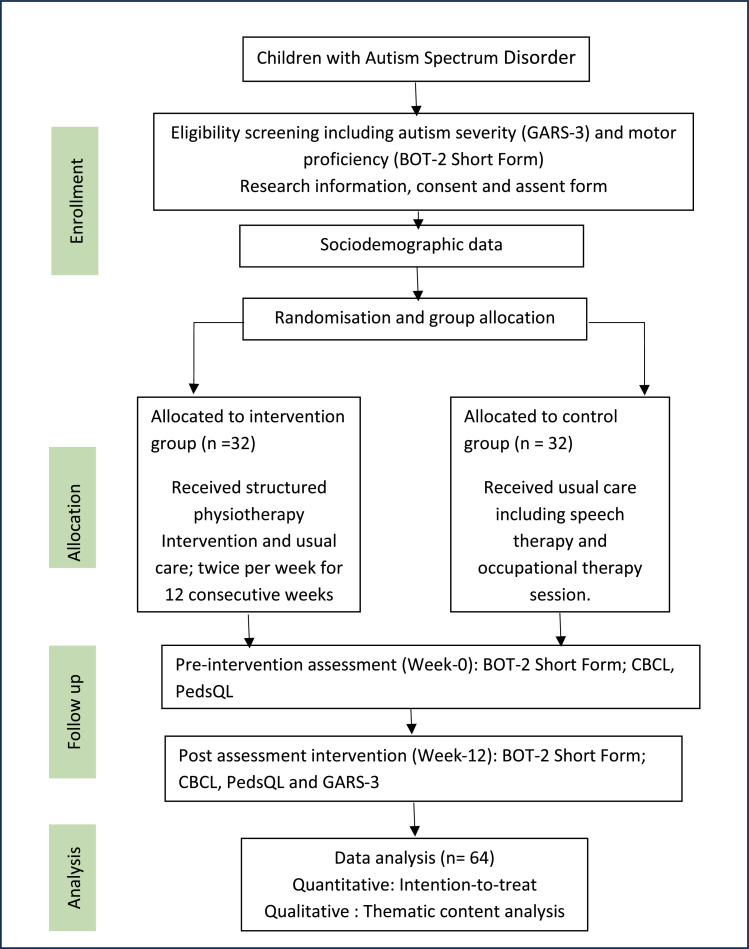
Flow chart of the study.

All children with ASD will be screened and recruited from the sampling frame of participating centres, using simple random sampling. The screening, recruitment, and randomisation procedures will be conducted by an independent researcher who is not involved in data collection or outcome assessment. Screening assessments of GARS-3 and BOT-2 Short Form will be conducted before randomisation to determine eligibility. As these assessments will be administered immediately before group allocation and under the same standardised conditions required for baseline measurement, the screening scores will be used as baseline data to minimise participant burden and avoid unnecessary repetition of measures. Eligible participants and their caregivers will receive clear, comprehensive information about the study through written materials and verbal explanations. This will include the study objectives, procedures, potential risks and benefits, and the anticipated time required for participation. After confirming their understanding, parents or legal guardians will be asked to provide written informed consent. Assent will also be obtained from the participating children in accordance with ethical standards for research involving minors. Only children classified as having mild to moderate ASD according to the GARS-3 will be invited to attend a motor skills assessment using BOT-2 Short Form. If a selected participant declines, the next name on the list will be contacted to achieve the desired recruitment target. Screening will take place over a two-week period, during which eligibility will be confirmed. Following confirmation of eligibility, participants will be randomised into either the intervention or control group using a computer-generated randomisation sequence (randomization.com). To reduce potential confounding, stratified randomisation will be applied by age group (6–8 years vs. 9–10 years) and ASD severity (mild vs. moderate, based on GARS-3). This approach will balance participant characteristics across study arms and ensure that outcomes reflect the intervention's effect independent of these variables. Allocation concealment will be maintained to prevent selection bias. Children who are successfully randomised will then be scheduled for baseline parent proxy assessments, conducted before the initiation of the intervention. GARS-3 and BOT-2 Short Form scores obtained during screening will serve as baseline values, while the GLTEQ, CBCL, and PedsQL will be administered as parent-proxy assessments following randomisation but before the intervention begins. All five outcome measures will be re-administered at post-intervention. This sequence ensures that group allocation is unbiased and that baseline data reflect participants’ pre-intervention status. A SPIRIT schedule of enrolment, interventions, and assessments is shown in Fig. 1.

### Blinding

A single-blind approach will be employed. Outcome assessors will remain blinded to group allocation to minimise bias in outcome ascertainment. Participants and therapists cannot be blinded due to the nature of the intervention. The success of assessor blinding will be assessed by asking the blinded outcome assessor to guess the participant’s group allocation as intervention, control or unsure after each assessment. Responses will be summarised descriptively and quantified using Bang’s Blinding Index [22].

### Intervention

Participants in the intervention group will receive a structured physiotherapy program in addition to their usual care. The sessions will be delivered by the main researcher (first author), a trained physiotherapist with >10 years of experience with children with ASD, using a standardised protocol to ensure consistency. Session checklists and corrective feedback logs will ensure fidelity.

Session Structure and Delivery: The structured physiotherapy programme will be delivered in small groups of 2 participants, with 2 60-minute sessions per week across 12 weeks (24 sessions). Each session follows a standardised structure: 5–8 min of warm-up, 40–45 min of core activities, and 5–7 min of cool-down and sensory regulation. Guided by the FITT (Frequency, Intensity, Time, Type) principle, the intervention integrates coordination, balance, strength, aerobic endurance and flexibility training progressively. Table 1 summarises the structured physiotherapy intervention. Neuromotor skills are addressed through bilateral integration and sequencing tasks; balance through static and dynamic challenges such as obstacle courses and beam walking; strength via age-appropriate resistance training (1–3 sets of 8–12 repetitions), aerobic endurance through game-based moderate-intensity activities and flexibility through dynamic stretches and yoga-inspired movements. The programme is delivered in three progressive phases: preparatory (weeks 1–4), development (weeks 5–8), and consolidation (weeks 9–12). Frequency involves two sessions per week. Intensity will progress from low to moderate (50–60 % HRmax) in the preparatory phase and gradually increase to moderate to vigorous (60–70 % HRmax) in the development and consolidation phases, depending on each child’s baseline motor abilities and tolerance. The whole sequence of activities is detailed in Table 1. Parents will be encouraged to participate in the sessions by observing and engaging with selected activities alongside their child. The parents’ involvement aims to reinforce motor learning, strengthen parent–child interaction, and facilitate continuity of practice through daily home activities.

**Table 1:** Summary of structured physiotherapy intervention.

Phase	Objectives	Components
Week 1–4 Preparatory Phase	Familiarisation with the strength, coordination, endurance and flexibility components in the training. Explore body segments; improve upper limb and lower limb coordination; low intensity training; breathing control	Warm-up: Mobility & breathingEndurance: Trampoline jumping, Figure-8 walkingStrength: Gymball lifts, Dumbbell raises, Wall squatsCoordination: Bear crawl, Arm circleBalance: Tandem walk, Tree pose
Week 5–8 Development Phase	Build strength, coordination and endurance. Develop unilateral strength, balance & symmetry; moderate intensity; breathing control	Warm-up: Light aerobicEndurance: Run + bean bag scrambleStrength: Medicine ball slams, Biceps curl, Frog jumpsCoordination: Catch/throw beanbagBalance: Beam walk
Week 9–12 Consolidation Phase	Develop strength, coordination, endurance and flexibility; focus on social-interaction task activity; high-intensity control; breathing control	Warm-up: TrampolineEndurance: Running + hurdlesStrength: Towel pull, BridgingCoordination: DribblingBalance: Single-leg variations

Assessment and Monitoring: Children’s heart rate will be monitored using a standard finger pulse oximeter, which provides real-time heart rate readings in addition to oxygen saturation. Age-predicted HRmax calculated using the formula: 220 – age [23]. Measurements will be taken at least twice during the aerobic component of each session, once at mid-point and once near the end, to ensure that activities remain within the targeted intensity range. In cases where children do not tolerate the pulse oximeter, intensity will be monitored using the OMNI Paediatric Perceived Exertion Scale and by observing behavioural indicators, such as breathing rate and speech ability. The measure will be recorded at each session to cross-validate intensity. Task progression is determined using a standardised 4-point technique checklist (1 = unable, 2 = requires assistance, 3 = independent with errors, 4 = independent with correct technique). The 4-point technique assessment is conducted by the treating physiotherapist during task performance and recorded on standardised forms for progression decisions. Progression occurs when the child achieves level 3–4 performance on ≥75 % of task attempts within a session. Progression made based on the guidelines provided by the American College of Sports Medicine 2020 [24]. Correct technique will be defined as the ability to perform ≥75 % of task repetitions with the intended movement pattern, posture and range of motion as demonstrated during training by the physiotherapist. If a child is unable to achieve this threshold, the activity will be regressed to a simpler form. For strengthening exercise, children will complete 1–3 sets of 8–12 repetitions, with resistance increased via bands or added complexity once children perform ≥75 % of tasks with correct technique. Aerobic activities will gradually increase from 8 to 15 min per session. Balance and coordination challenges will advance by reducing external support, increasing speed, or adding dual-task elements. Short breaks are provided between tasks to accommodate individual needs. If a child refuses to perform an activity, the session proceeds to the next task, and all completed activities are recorded.

Group Management Strategy: Within each 4-week phase, the same core activities are maintained to allow skill consolidation, but their complexity is progressively increased based on individual performance. Progression occurs when a child demonstrates ≥75 % correct technique, regardless of the week within the phase. One example is during the preparatory phase (weeks 1–4), all children practice the same core balance activity (single-leg stance). However, progression occurs individually: starting with eyes open/arms free, advancing to eyes closed, then adding dual tasks, regardless of which week within the phase. New activity types are only introduced in subsequent phases. Each child’s progression will be individualised based on their ability to perform tasks with correct technique and tolerance. If one child progresses more rapidly than another, the physiotherapist will adjust the session by maintaining the same activity theme while assigning tasks at different levels of difficulty. These adjustments may involve modifying parameters such as distance, load, or balance demands (e.g., shorter versus longer distances, lighter versus heavier balls, or wider versus narrower balance paths). For instance, when implementing the balance beam activity for individualised progression and regression in a group setting, different strategies can be applied based on each child's skill level. For children who have not achieved at least 75 % correct technique, regression strategies include widening the beam, reducing walking speed, and simplifying the task to forward walking only. Conversely, for those who meet the progression threshold, the task can be advanced by narrowing the beam width, increasing walking speed, and introducing a dual-task element, such as carrying an object while walking across the beam. This approach helps maintain group cohesion while providing an appropriate level of challenge for each child. Activities will be delivered in pairs, allowing for social interaction while accommodating differences in ability [25]. This paired approach ensures that children benefit from the motivational and social aspects of group participation while receiving individualised progression appropriate to their developmental level [25]. The physiotherapist will supervise both children simultaneously, ensuring safety, engagement, and consistency of delivery.

### Control group

Participants in the control group will continue with usual care only, defined as the range of therapeutic services accessible through routine clinical pathways at the participating centres. These typically include occupational therapy focusing on sensory integration and fine motor activities, speech-language therapy addressing social communication and pragmatic language goals, and clinical psychology sessions targeting behavioural regulation. In routine clinical practice, the frequency of these services varies according to service availability and individual care plans. Occupational therapy is commonly delivered on a monthly basis, while speech-language therapy and clinical psychology are typically provided at longer intervals, often approximately once every six months. The specific content and frequency of usual care may therefore vary across participants and centres, reflecting the heterogeneity of routine clinical practice. The frequency and intensity of these therapies will be recorded using standardised caregiver logs and verified with site records. During the 12-week study period, no additional structured motor-based intervention will be introduced. To minimise contamination, participants will be asked not to enrol in new motor-based programmes, and site clinicians will be informed of this restriction. A usual-care control group was selected to reflect real-world practice and enhance the external validity of the trial findings.

### Outcome measures

The primary outcome of this study is motor skill level. Motor skills will be measured using the BOT-2 Short Form, which is widely recognised as a comprehensive and standardised tool for assessing both fine and gross motor skills in children [26]. BOT-2 Short Form measures 4 motor area composites with 8 subtests comprised of 14 items. The BOT-2 Short Form includes subtests covering manual coordination, body coordination, strength, and agility. Standard scores will be calculated using age-specific norms, with higher scores indicating better motor performance. The BOT-2 has demonstrated strong reliability and validity in populations with ASD [27].

Secondary outcomes include autism symptoms, physical activity levels, behavioural problems, and QoL. Physical activity levels will be measured using the Godin–Shephard Leisure-Time Exercise Questionnaire (GLTEQ), a validated self-report tool adapted for proxy reporting by parents in paediatric populations [28]. The GLTEQ assesses the frequency of mild, moderate, and vigorous leisure-time physical activity over a typical 7-day period. The questionnaire comprises four items, requires approximately 10–15 min to complete, and yields categorical classifications: ≥24 units = Active; 14–23 units = Moderately Active; < 14 units = Insufficiently Active/Sedentary. A total leisure activity score is derived by weighting responses according to intensity, with higher scores indicating greater engagement in physical activity. Previous studies have confirmed GLTEQ’s validity and reproducibility in monitoring physical activity among children with ASD [29].

Autism symptoms and severity will be assessed using the GARS-3, a validated tool for assessing autism-related behaviours in children [30]. The scale includes 58 items across six subscales: Restricted/Repetitive Behaviours, Social Interaction, Social Communication, Emotional Responses, Cognitive Style and Maladaptive Speech [30]. Items are rated on a 4-point frequency scale to generate subscale scores and an overall autism index, with higher scores indicating greater severity [30]. The GARS-3 demonstrates strong psychometric properties and is widely used in both clinical and research settings [30].

Behavioural problems will be evaluated using the Child Behaviour Checklist (CBCL), a parent-reported questionnaire designed to evaluate emotional and behavioural functioning in children [31]. The CBCL includes 118 items, with responses rated on a 3-point scale (“Not True”, “Somewhat or Sometimes True”, “Very True or Often True”) [31]. Composite scores will be generated for internalising problems, externalising problems, and total behaviour problems. The CBCL has been widely validated internationally, including in ASD populations, and provides a robust measure of behaviour-related changes following intervention [32].

Quality of life will be assessed using the Paediatric Quality of Life Inventory (PedsQL), which measures health-related quality of life across four domains: physical, emotional, social, and school functioning [33]. The 23-item parent-proxy form will be administered, with responses rated on a 5-point Likert scale from “Never” to “Almost Always”. Items are reverse-scored and linearly transformed to a 0–100 scale, with higher scores reflecting better QoL [33]. The PedsQL has strong psychometric properties and has been validated for the ASD population [34].

### Semi-structured focus group discussion

A qualitative component was incorporated in this study to obtain a deeper understanding of the perceived effectiveness of the structured physiotherapy intervention. A post-intervention focus group discussion (FGD) will be conducted among parents and caregivers whose children completed the 12-week programme. The FGD aimed to explore caregivers’ experiences, perceived changes in their child’s motor skills, physical activity levels, behaviour, and overall QoL following the intervention. Key discussion prompts included perceptions of motor skills levels, noticeable differences between usual care and the structured physiotherapy sessions, the acceptability and practicality of the intervention, and the perceived value of continuing the programme. The semi-structured FGD interview protocol including main questions and probing cues is presented in Table S1. The FGD will be conducted a week after completion of all post-intervention assessments and will last approximately 45–60 min. This FGD provided contextual insights that complemented quantitative findings and informed the interpretation of the intervention’s real-world relevance and feasibility.

### Data analysis

Data will be analysed using IBM SPSS Statistics Version 28. Descriptive statistics will summarise participant characteristics, and baseline comparability between groups will be evaluated using independent *t*-tests or chi-square tests. All analyses will follow the intention-to-treat (ITT) principle, with missing data handled using last observation carried forward (LOCF) and will be supported by sensitivity analyses using multiple imputation. A 2 × 2 mixed-design ANOVA (group × time) will examine within and between-group differences across the intervention period. Significant interactions will be examined using Bonferroni-adjusted post hoc tests. Effect sizes will accompany all significance tests, including Cohen’s d for mean differences and partial eta squared (η²p) for ANOVA effects, with 95 % confidence intervals reported. Statistical significance will be set at *p* < 0.05. Qualitative data will be analysed using thematic analysis following Braun and Clarke’s six-phase protocol [35].

### Safety and adverse events

The structured physiotherapy intervention is low risk, consisting of age-appropriate, motor-based intervention delivered by qualified physiotherapists. Potential risks include minor fatigue, muscle soreness, or reduced motivation to participate. To mitigate these risks, sessions will incorporate warm-up and cool-down routines, provide adequate breaks between tasks, and allow children to decline activities they do not wish to perform. All activities will be supervised closely by trained therapists. Any adverse events will be documented and reported to the ethics committee.

### Data confidentiality

All data collected in this study will be stored in password-protected electronic files accessible only to the research team. Identifiable information, such as names and contact details, will be kept separately from study data to maintain confidentiality. Data will be retained in accordance with institutional guidelines and national regulations, and results will be reported in aggregate form without identifying individual participants.

### Strengths and limitations

This study has notable strengths, including the use of a structured physiotherapy intervention grounded in established motor and behavioural frameworks, as well as a mixed-methods approach that integrates quantitative outcomes with qualitative insights. However, several limitations should be considered. Parent-reported measures such as the PedsQL, CBCL, and GLTEQ may be influenced by subjective perceptions or social desirability. Usual-care practices in the control group may vary across centres and therapists, introducing unavoidable heterogeneity. Additionally, the sample is limited to children aged 6–10 years with mild to moderate ASD, which restricts generalizability to other age groups or severity levels. Findings should therefore be interpreted within the context of this defined population.

### Ethics and dissemination

This study is approved by the National University of Malaysia’s Human Research Ethics Committee (JEP-2024–1127). This study will be conducted in accordance with the Good Clinical Practice Guideline and the ethical principles of the Declaration of Helsinki 1964. Results will be disseminated through peer-reviewed journals and international conferences.

## Protocol validation

The clinical study associated with this protocol is ongoing, with participant recruitment and intervention delivery underway. No data are available at this stage to support protocol validation as outcome assessments have not yet commenced.

## Declaration of generative AI and AI-assisted technologies in the manuscript preparation process

During the preparation of this work, the author(s) used ChatGPT (OpenAI) to have language refinement and clarity. After using this tool/service, the author(s) reviewed and edited the content as needed and take(s) full responsibility for the content of the published article.

## CRediT authorship contribution statement

**Nazurah Alwi:** Project administration, Writing – original draft, Writing – review & editing. **Asfarina Zanudin:** Conceptualization, Funding acquisition, Supervision, Writing – review & editing. **Mahadir Ahmad:** Conceptualization, Supervision, Writing – review & editing. **Nor Azizah Mohamad:** Conceptualization, Supervision.

## Declaration of interests

The authors declare that they have no known competing financial interests or personal relationships that could have appeared to influence the work reported in this paper.

## Data Availability

No data was used for the research described in the article.

## Appendix Supplementary materials

Image, application 1
