# Supplementary material for: Effectiveness of a structured physiotherapy intervention on psychomotor and quality of life in children with autism spectrum disorder: A randomised controlled trial protocol
Source: MethodsX. 2026 Mar 18;16:103873. doi: 10.1016/j.mex.2026.103873 (PMC13059026; doi:10.1016/j.mex.2026.103873)
Supplement: Supplementary file 1 [file mmc1.docx]

**Supplementary material *and/or* additional information**

Table SPIRIT schedule of enrolment, interventions, and assessments

| Timepoint | Enrolment | Allocation | Baseline (T1) | Post-intervention (12 weeks) (T2) |
| --- | --- | --- | --- | --- |
| **Enrolment** |  |  |  |  |
| Eligibility screen | X |  |  |  |
| Informed consent | X |  |  |  |
| Allocation |  | X |  |  |
| **Interventions** |  |  |  |  |
| Intervention group: Structured physiotherapy intervention (FITT-based, 24 sessions over 12 weeks) |  |  |  |  |
| Control group: Usual therapy and physical activities |  |  |  |  |
| **Assessments** |  |  |  |  |
| Sociodemographic data | X |  |  |  |
| Motor skills (BOT-2) | X |  |  | X |
| Autism symptoms (GARS-3) | X |  |  | X |
| Physical activity (GLTEQ) |  |  | X | X |
| Behavior (CBCL) |  |  | X | X |
| Quality of life (PedsQL) |  |  | X | X |

Table Semi-Structured Focus Group Discussion (FGD) Interview Protocol

| Domain Measure | Interview Questions and Probes |
| --- | --- |
| Motivation and Expectations | What motivated you to enrol your child in this physiotherapy programme and what were your initial expectations?  Probe: reasons for joining, goals, prior therapy experiences |
| Motor Skill Changes | What changes have you noticed in your child’s movement abilities since the programme began?  Probe: balance, coordination, strength, endurance, daily movement tasks |
| Physical Activity Participation | How has your child’s involvement in physical activities or play changed since completing the programme?  Probe: type of activities, frequency, duration, intensity of play |
| Emotional Regulation | How would you describe any changes in your child’s emotions or mood since participating in the programme?  Probe: emotional expression, confidence, anxiety, irritability, self-regulation |
| Behavioural Patterns | In what ways, if any, have your child’s behavioural patterns changed since completing the programme?  Probe: repetitive behaviours, turn-taking, following instructions, challenging behaviours |
| Social Interaction and Communication | How has your child’s way of interacting and communicating with others changed since the programme?  Probe: peer engagement, verbal/non-verbal communication, group settings, responsiveness |
| Overall Quality of Life | How has the programme affected your child’s daily life and your family’s overall quality of life?  Probe: school participation, daily routines, sleep, independence, family stress |
| Child Engagement | How did your child respond to the physiotherapy sessions throughout the 12 weeks?  Probe: favourite/least favourite activities, motivation over time, ease of attendance |
| Comparison with Usual Care | How would you compare the structured physiotherapy sessions with the other therapies your child receives?  Probe: perceived differences, added value, unique contributions of physiotherapy |
| Barriers and Challenges | What challenges did you and your child face during the programme, and how did you manage them?  Probe: scheduling, transportation, child behaviour, specific exercise difficulties, family commitment |
| Perceived Effectiveness and Recommendations | Looking back at the entire programme, what aspects were most helpful for your child, and what would you suggest to improve it?  Probe: most/least effective components, schedule changes, parental involvement, recommendations for future |
